# Poly-L-Lysine–Lactobionic Acid-Capped Selenium Nanoparticles for Liver-Targeted Gene Delivery

**DOI:** 10.3390/ijms23031492

**Published:** 2022-01-27

**Authors:** Shaigan Naidoo, Aliscia Daniels, Saffiya Habib, Moganavelli Singh

**Affiliations:** Nano-Gene and Drug Delivery Group, Discipline of Biochemistry, School of Life Sciences, College of Agriculture, Engineering and Science, University of KwaZulu-Natal (Westville Campus), Private Bag X54001, Durban 4000, South Africa; 215037110@stu.ukzn.ac.za (S.N.); DanielsA@ukzn.ac.za (A.D.); Saffiya.habib@gmail.com (S.H.)

**Keywords:** selenium nanoparticles, poly-L-lysine, lactobionic acid, hepatocellular carcinoma, nanomedicine, gene expression

## Abstract

Liver cancer is currently regarded as the second leading cause of cancer-related mortality globally and is the sixth most diagnosed malignancy. Selenium nanoparticles (SeNPs) have attracted favorable attention as nanocarriers for gene therapy, as they possess beneficial antioxidant and anticancer properties. This study aimed to design, functionalize and characterize SeNPs to efficiently bind, protect and deliver pCMV–*Luc* DNA to hepatocellular carcinoma (HepG2) cells. The SeNPs were synthesized by ascorbic acid reduction and functionalized with poly-L-lysine (PLL) to stabilize and confer positive charges to the nanoparticles. The SeNPs were further decorated with lactobionic acid (LA) to target the asialoglycoprotein receptors abundantly expressed on the surface of the hepatocytes. All SeNPs were spherical, in the nanoscale range (<130 nm) and were capable of successfully binding, compacting and protecting the pDNA against nuclease degradation. The functionalized SeNP nanocomplexes exhibited minimal cytotoxicity (<30%) with enhanced transfection efficiency in the cell lines tested. Furthermore, the targeted SeNP (LA–PLL–SeNP) nanocomplex showed significant (* *p* < 0.05, ** *p* < 0.01, **** *p* < 0.0001) transgene expression in the HepG2 cells compared to the receptor-negative embryonic kidney (HEK293) cells, confirming receptor-mediated endocytosis. Overall, these functionalized SeNPs exhibit favorable features of suitable gene nanocarriers for the treatment of liver cancer.

## 1. Introduction

Liver cancer has the second-highest cancer-related mortality rate worldwide. It comprises heterogeneous groups of malignant tumors, which include hepatocellular carcinoma (HCC), intrahepatic cholangiocarcinoma (iCCA), mixed hepatocellular cholangiocarcinoma (HCC-CCA), fibrolamellar HCC (FLC) and paediatric neoplasm hepatoblastoma [1,2]. HCC is a primary liver cancer that stems from hepatocytes and accounts for 90% of all primary liver cancers [3]. It is expected that over a million people will be affected annually by the year 2025 [4]. Several pathways may be affected by HCC, including oxidative stress and detoxifying pathways, metabolism of iron pathways and DNA repair mechanisms [5,6]. Current cancer treatments include surgery, radiation therapy and chemotherapy. However, these treatments have had minimal influence on reducing cancer mortality and become less effective if the tumor cells metastasize to other parts of the body [7]. Furthermore, there are multiple obstacles associated with cancer treatments, including tumour heterogeneity, drug resistance and systematic toxicities throughout the body [8,9]. 

A promising form of treatment is gene therapy, which is used to correct or improve the symptoms of a disease by introducing an exogenous gene into cells that may modify a defective gene or initiate cell death. Gene therapy requires an efficient, safe and specific delivery system with a high gene insertion capacity and transfection rate and an administration method that is noninvasive or harmful [10]. Inorganic nanoparticles are promising prospects as nonviral vectors. They possess numerous beneficial properties for cellular gene delivery, including efficient biocompatibility, storage stability, ease of preparation, wide availability, the potential of targeted delivery [11] and low cytotoxicity [12]. Interactions between nanoparticles (NPs) and biomolecules are essential for the successful loading and cellular transfection. Furthermore, for gene and drug delivery to achieve the desired success, novel strategies and pharmaceutical drug leads need to be developed [13]. Nanomaterials in the the form of NPs, nanofibers [14] and spindles [15] have been produced form organic and inorganic material. Several NPs have been utilized to date and have been generally classified as organic, carbon-based or inorganic NPs [16]. Among the various inorganic NPs such as gold, silver, platinum, selenium and mesoporous silica that have been researched to date, selenium nanoparticles (SeNPs) have shown great potential in nanomedicine. 

SeNPs have displayed increased biocompatibility and bioavailability that has matched other inorganic delivery vectors [17,18]. They possess low toxicity compared to various selenium compounds and exhibit potential therapeutic and diagnostic roles, thus making SeNPs likely elements for applications in clinical and biomedical fields [19,20,21,22,23,24]. SeNPs have beneficial biological properties compared to inorganic and organic selenium compounds, as they have greater efficiency as a cofactor for thioredoxin reductase and glutathione peroxidase [21,25,26,27]. In addition to Se being an essential micronutrient, it is critical to normal body function, and its supplementation to treat various diseases have been recorded [22]. Owing to these benefits, the application of SeNPs to liver-directed gene therapy may be a viable option, considering the possibility of synergistic therapeutic effects using SeNPs as delivery agents. SeNPs have been recently reported for the liver-directed delivery of mRNA [28].

Uncoated SeNPs lack stability, which could affect the physicochemical characteristics of the NP [29]. Surface modifications with a cationic surfactant allow for electrostatic interactions between an NP and a negatively charged biomolecule, e.g., DNA. Loading of the biomolecule is dependent on the charge density, modifier structure and length of the organic chain. The modifier may also protect the bound biomolecule [26]. The SeNPs used in this study were functionalized and stabilized with poly-L-lysine (PLL). Lysine possesses attractive properties such as biocompatibility, biodegradability, hydrophobicity [30], low toxicity and no antigenicity [31]. Lysine residues bind to the surface of the SeNPs via electrostatic interactions due to the presence of two NH_3_^+^ groups, providing greater stability and a cationic surface [32]. Furthermore, the exposed NH_3_^+^ groups on lysine residues allow electrostatic interaction and complex formation with nucleic acids [19]. This makes PLL a suitable surface modification of inorganic NPs for gene delivery.

Cell specificity can be achieved by introducing a cell recognition component onto the NP surface, allowing the NP to enter the cell via receptor-mediated endocytosis (RME). Active targeting is accomplished by attaching targeting ligands on the surface of the NPs, enabling the NPs to bind to desired cognate receptors that are over-expressed on the target tumor cells and not expressed on normal cells [33,34]. The asialoorosomucoid receptor (ASGP-R) abundantly found on the surface of hepatocytes has a high affinity for ligands that contain terminal galactose residues [35]. For efficient targeting of the asialoorosomucoid receptor (ASGP-R), the PLL–SeNPs was further modified with the galactose containing moiety, lactobionic acid (LA). The LA was conjugated onto the amino group of PLL via EDC/NHS coupling, followed by the introduction of the synthesized SeNPs to the newly formed LA–PLL coating, producing targeted LA–PLL–SeNPs. LA had been used successfully as a liver-targeting ligand in recent studies [28,36].

The use of PLL–SeNPs as nonviral gene delivery vehicles that can bind and protect their genetic cargo and bring about significant transgene expression has not been fully explored. Hence, this proof of principle study was designed to utilize these LA–PLL–SeNPs as liver-targeted delivery vehicles of the pCMV–*Luc* DNA reporter gene. 

## 2. Results

### 2.1. Nanoparticle Synthesis and Characterization 

**Figure 1:**
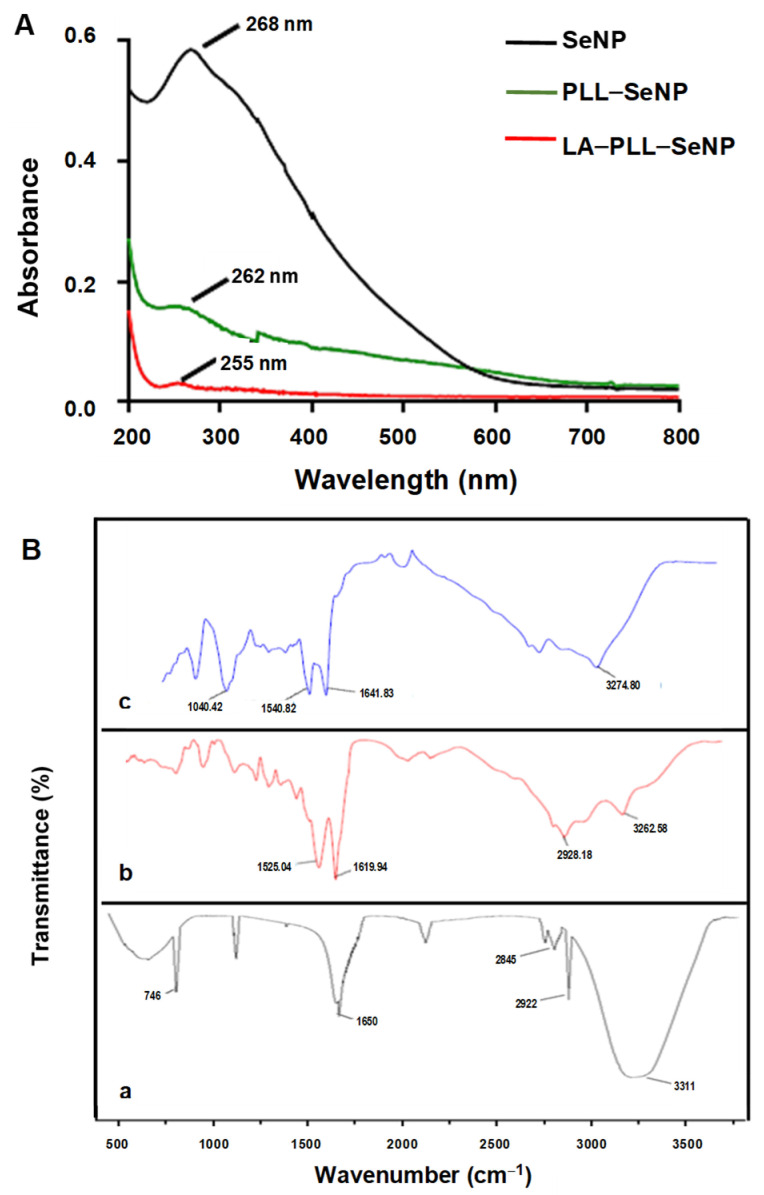
(**A**) UV-vis spectra of SeNP, PLL–SeNP and LA–PLL–SeNP; (**B**) FTIR spectra of (a) SeNP, (b) PLL–SeNP and (c) LA–PLL–SeNP.

The formation and functionalization of the SeNPs were first visually confirmed, with SeNPs appearing orange and the PLL-functionalized SeNPs displaying the characteristic red color, as reported previously [37]. UV-vis and FTIR spectroscopy were further utilized to confirm the synthesis and functionalization of the SeNPs. Figure 1A shows the UV-vis spectra of the synthesized NPs. All NPs possessed characteristic peaks with the SeNPs exhibiting a λmax at 268 nm. PLL–SeNP had a λmax at 262 nm, and LA–PLL–SeNP had a λmax at 255 nm, indicating a blue shift from the original SeNP upon each modification. Notably, the absorbance of the FSeNPs was much lower than that of the SeNPs. 

FTIR reflected the characteristic bands for the SeNPs and FSeNPs (Figure 1B). The uncoated SeNPs (a) exhibited peaks at 3311 cm^−1^, accounting for the hydroxyl group (−OH); two sharp peaks at 2845 cm^−1^ and 2922 cm^−1^; indicating C–H symmetric and asymmetric stretching respectively; and a peak at 1650 cm^−1^, indicating the amide I of α-helical structures [38]. A broad absorption peak was observed for PLL–SeNP (b) at 2928.18 cm^−1^, indicating C–H stretching [39]; at 3262.58 cm^−1^, indicating an amide A; and two peaks at 1619.94 and 1525.04 cm^−1^ corresponding to a β-sheet conformation of PLL on the SeNP surface [40]. For the LA–PLL–SeNPs (c), there was a redshift in the amide A absorption peaks from 3262.58 cm^−1^ to 3274.80 cm^−1^, as well as the for the amide I and II absorption peaks from 1619.94 and 1525.04 cm^−1^ to 1641.83 and 1540.82 cm^−1^, respectively. A distinct peak at 1040.42 cm^−1^ indicated C−O stretching. This confirmed LA–PLL–SeNP synthesis.

**Figure 2:**
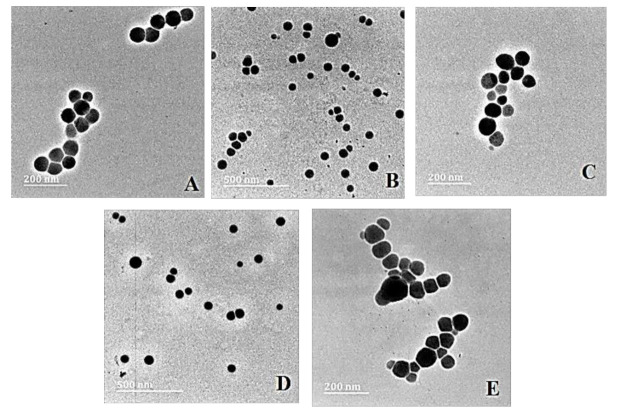
TEM images of (**A**) SeNP, (**B**) PLL–SeNP, (**C**) LA–PLL–SeNP, (**D**) pDNA–PLL–SeNP and (**E**) pDNA–LA–PLL–SeNP. Scale Bar = 200 nm (**A**,**C**,**E**) and 500 nm (**B**,**D**).

Transmission electron microscopy (TEM) provided the ultrastructural characteristics of the NPs and their nanocomplexes with pDNA, revealing spherical particles with no significant agglomeration (Figure 2). 

Nanoparticle tracking analysis (NTA) was used to determine the size, zeta potential (ζ) and polydispersity of the NPs and nanocomplexes. The results (Table 1) confirmed that the NPs and their nanocomplexes fell within the nanometer range (0–200 nm), which is considered a desirable feature for the use of NPs in nanomedicine [41,42]. The zeta potentials obtained alluded to NPs and nanocomplexes with moderate to good stability. The PDI values were all below 0.1, suggesting a monodisperse population. 

### 2.2. Electrophoretic Mobility Shift Assay

**Figure 3:**
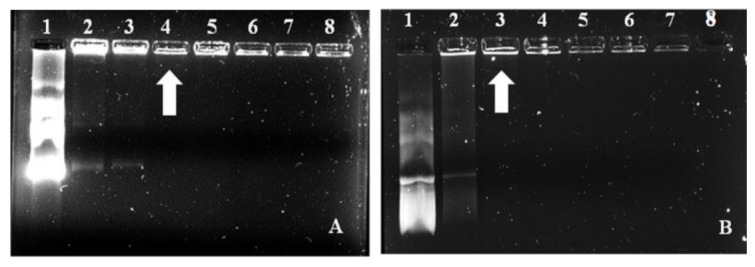
Electrophoretic mobility shift assay of FSeNP:pDNA nanocomplexes. Lane 1: pDNA control = 0.25 μg. Lanes 2–8: 0.25 μg pDNA complexed to varying amounts of FSeNPs (μg/μL) as follows: (**A**) PLL–SeNP (w/w) (0.45; 0.55; 0.67; 0.8; 0.9 and 1) and (**B**) LA–PLL–SeNP (w/w) (2.73; 6.23; 4.1; 1:4.88; 5.55 and 6.23). Arrows indicate the optimum binding ratio of the FSeNP:pDNA.

This assay was used to determine the amount of the FSeNP needed to bind 0.25 μg/μL pDNA. The results of the electrophoretic mobility or band shift assay are represented in Figure 3, with the corresponding binding ratios (optimal, suboptimal supraoptimal binding ratios of the FSeNPs:pDNA) being depicted in Table 2. The results show that both PLL–SeNP and LA–PLL–SeNP could efficiently bind the pDNA at relatively low concentrations. 

### 2.3. Dye Displacement Assay

**Figure 4:**
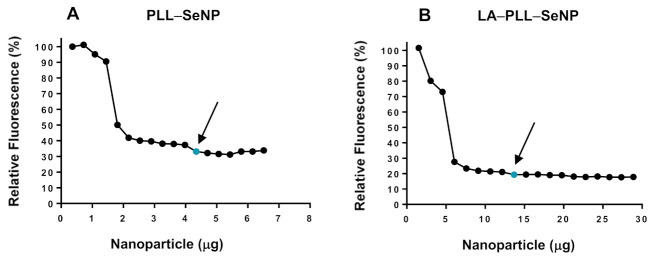
Dye displacement assay of (**A**) PLL–SeNP and (**B**) LA–PLL–SeNP. The arrows and blue dots indicate points of inflection.

The dye displacement assay was conducted to assess the FSeNPs ability to condense pDNA by monitoring the decay of ethidium bromide (EB) with increasing amounts of the NP. This assay used the pDNA:EB mixture as 100% fluorescence. The addition of the FSeNPs resulted in the displacement of the intercalated EB and a concomitant reduction in the fluorescence. The fluorescence decreased until it reached a plateau, indicating maximum compaction of pDNA by each NP. The fluorescence quenching displayed by PLL–SeNP and LA–PLL–SeNP (Figure 4A,B), provide evidence that these FSeNPs can bind, condense and compact the pDNA. The targeted NPs (LA–PLL–SeNPs) showed a greater compaction potential with a fluorescence decay of 80.7%, compared to the untargeted NPs (PLL–SeNPs) with a fluorescence decay of 66.8%. 

### 2.4. Enzyme Protection Assay 

**Figure 5:**
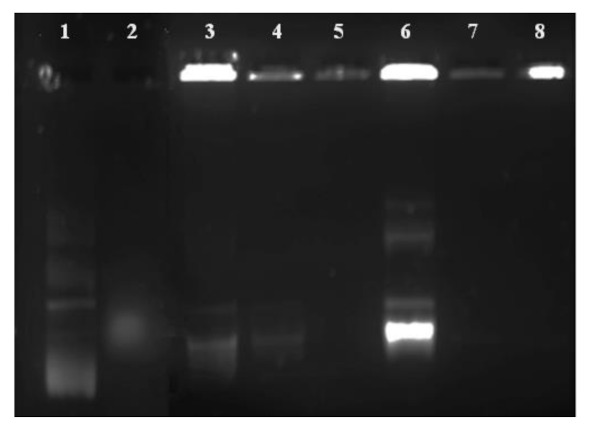
Enzyme protection assay of pCMV–*Luc* DNA containing nanocomplexes. Lane 1: positive control (untreated pDNA). Lane 2: negative control (pDNA + 10% FBS). Lanes 3–5: PLL–SeNP nanocomplexes with pDNA (w/w) (1:2.2; 1:2.7; 1:3.2). Lanes 6–8: LA–PLL–SeNP nanocomplexes with pDNA (w/w) (1:10.9; 1:14; 1:16.4).

Protection from nuclease degradation is an essential parameter for the successful delivery of pDNA by the FSeNPs [43]. To assess the FSeNPs’ ability to protect the pDNA, the enzyme protection assay using serum nucleases was performed to simulate in vivo conditions. The agarose gel is presented in Figure 5. The positive pDNA control (lane 1) displayed the migration of its characteristic bands. In contrast, the negative control (lane 2) produced a smear with the absence of distinct bands indicating degradation of the pDNA. Overall, the FSeNPs were able to protect the pDNA from enzyme degradation. Although sodium dodecyl sulphate (SDS) was used to release the pDNA from the nanocomplex, much of the pDNA remained well-bound.

### 2.5. MTT Cell Viability Assay 

**Figure 6:**
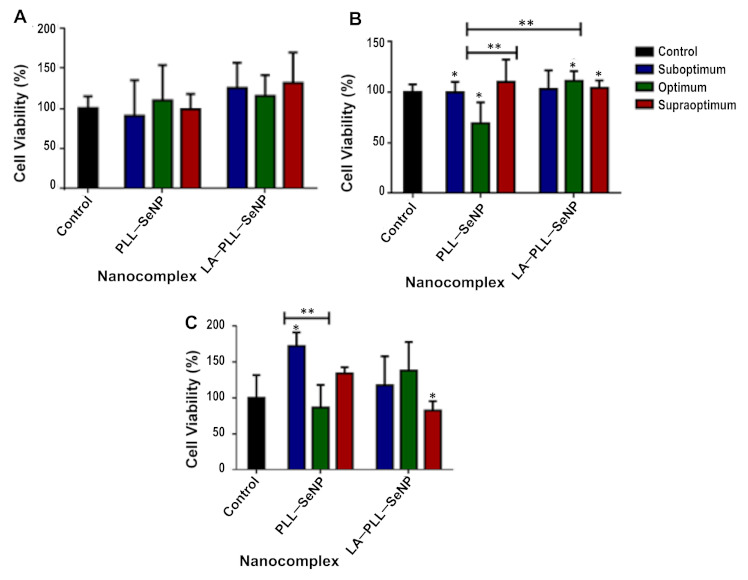
Cytotoxicity profiles of the FSeNP nanocomplexes in (**A**) HEK293, (**B**) HeLa and (**C**) HepG2 cell lines. The control represents untreated cells and 100% cell viability. Data are represented as mean ± SD (*n* = 3). * *p* < 0.05 and ** *p* < 0.01 show statistical significance within each nanocomplex and between ratios of both nanocomplexes (untargeted vs. targeted).

The cytotoxic effects of the FSeNP nanocomplexes were evaluated on the cancer cell lines, HeLa and HepG2, and on the noncancer cell line, HEK293, using the MTT assay. This assay is based on the principle that only viable cells can reduce the tetrazolium dye, 3-(4,5dimethylthiazol-2-yl)-2,5-diphenyltetrazolium bromide using mitochondrial dehydrogenases to produce purple formazan crystals found in the mitochondria, cytoplasm and even the plasma membrane [8]. These crystals are then solubilized in DMSO and quantified spectrophotometrically to create the cytotoxicity profiles for each nanocomplex, as shown in Figure 6. 

All cell lines treated with modified SeNPs displayed no significant cytotoxicity with greater than 69% cell viability. The HepG2 cells had less than 20% cytotoxicity, followed closely by the HEK293 cells.

### 2.6. Apoptosis

**Figure 7:**
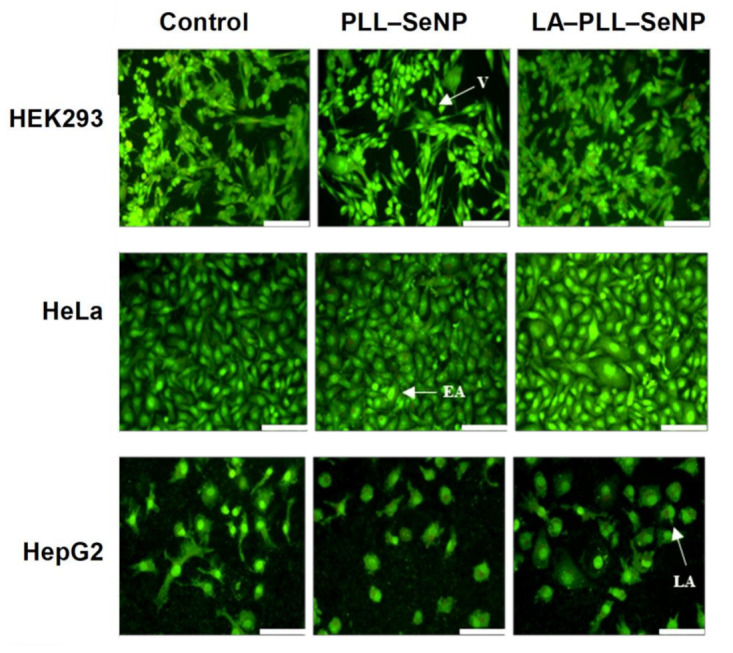
Fluorescent images obtained from AO/EB apoptosis studies after treatment with nanocomplexes in the HEK293, HeLa and HepG2 cells at 20× magnification. Scale bar = 100 μm. V—viable cells, EA—early apoptotic cells, LA—late apoptotic cells.

Since results from the MTT assay revealed that the FSeNP formulations induced minimal cytotoxicity in the cell lines tested, the acridine orange/ethidium bromide (AO/EB) dual staining apoptosis assay was used to determine if there existed any correlation between cell death, apoptosis and necrosis. The AO penetrates all cells and emits a green fluorescence, that indicates healthy cell nuclei. In contrast, the EB dye pervades cells with a compromised cytoplasmic membrane, and thus, a yellow-to-red fluorescence is emitted [44,45]. The fluorescent images (Figure 7) and apoptotic indices (Table 3) provided evidence that the FSeNP formulations produced negligible apoptosis at their optimum binding ratios, as evidenced by most cells appearing green, which indicated viable cells. Importantly, no necrotic cells were observed in any of the cells.

### 2.7. Reporter Gene and Receptor Competition Assay

**Figure 8:**
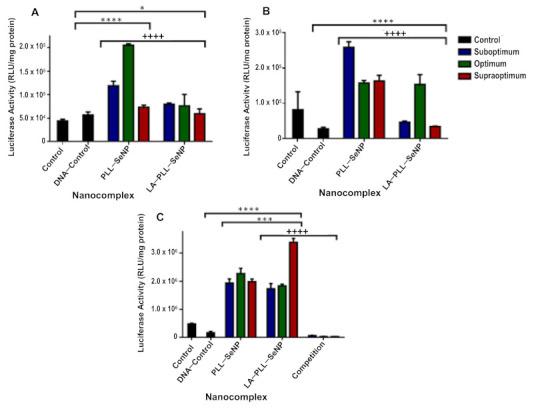
Transfection activity of FSeNP nanocomplexes in (**A**) HEK293, (**B**) HeLa and (**C**) HepG2 cell lines. Control: untreated cells. DNA control: cells treated with naked pDNA (0.25 μg/μL). Data are presented as means ± SD (*n* = 3). * *p* < 0.05 and **** *p* < 0.0001 show statistical significance between DNA control and FSeNP nanocomplexes. *** *p* < 0.001 shows statistical significance between untargeted vs. targeted nanocomplexes. ^++++^ *p* < 0.0001 shows statistical significance between targeted nanocomplexes vs. targeted nanocomplexes in competition assay. The competition assay (**C**) shows the transfection activity of LA–PLL–SeNP nanocomplexes in the HepG2 cells after blocking the ASGP-R.

The luciferase activity obtained for the FSeNP nanocomplexes are shown in Figure 8. It is evident that these nanocomplexes exhibited a significant increase in luciferase activity (*p* < 0.0001) across all cell lines compared to the DNA control. PLL–SeNP overall showed enhanced expression in all the cell lines. However, LA–PLL–SeNP had the highest transgene expression in the HepG2 cells (Figure 8C) at the supraoptimal binding ratio, suggesting this ratio was ideal for cellular uptake via RME via the targeting moiety, LA. 

The competition binding study further confirmed the active targeting capabilities of the targeted nanocomplexes. As seen in Figure 8C, the luciferase activity was significantly decreased (*p* < 0.0001) when receptors on the HepG2 cells were blocked with excess LA. This decrease was noted at all ratios of the targeted nanocomplexes, with a 122-fold reduction in luciferase activity noted for the supraoptimum ratio of the targeted nanocomplexes, which displayed the highest luciferase activity. A 27-fold decrease was noted for the suboptimum ratio, which showed the lowest luciferase activity. These results confirm that the ASGP-R on the HepG2 cells recognized the targeted LA–PLL–SeNP:pDNA nanocomplex via the LA ligand conjugated to the nanocomplex.

## 3. Discussion

An ascorbic acid reduction successfully synthesized the SeNPs. Ascorbic acid’s biocompatibility and good reducing ability resulted in the formation of spherical NPs, with lower toxicity than that achieved using other reducing agents [46]. The SeNPs were modified and stabilized with the cationic polymer, poly-L-lysine (PLL), which also conferred positive charges, and the ligand lactobionic acid (LA), which facilitated targeting of the asialoorosomucoid receptor (ASGP-R), over-expressed on hepatocytes (HepG2 cells). UV-vis spectroscopy confirmed the presence of the SeNPs (λmax = 268 nm) [28] with the PLL and LA modified SeNPs displaying a blueshift in the spectrum, in addition to a drop in the absorbance. Capping agents influence the SPR, as it is the first material encountered on the NP. This encapsulation affects the electron oscillations around the NP, yielding variations in the SPR band [39], as evidenced for the functionalized SeNPs.

NTA provides an insight into the NP’s potential to bind and compact the pDNA and their suitability as gene delivery vehicles. The NPs were below 125 nm while the nanocomplexes were below 170 nm in size, suggesting their potential for use as delivery vehicles since NPs below 200 nm have been reported as favorable delivery systems [41,42]. Liver-directed lipid-based delivery systems around 141 nm in diameter have shown targeted gene expression in parenchymal cells in vivo. In comparison, larger systems (>200 nm) seemed to achieve good gene expression in non-parenchymal cells [47]. Furthermore, complexes >150 nm are restricted from traversing the liver tissue’s sinusoidal fenestrae [48]. Hence, it is vital to reduce the size of the nanocomplexes to achieve enhanced ASGP-R-mediated targeting [49]. The colloidal stability and the surface charge of the NPs are represented by the ζ potential [41]. NPs with a ζ potential that falls within a range of <−25 mV and >+25 mV are considered to be colloidally stable [50], boding well for in vivo applications. The addition of PLL to the SeNP surface resulted in greater stability of the NP with an increased ζ potential. However, the inclusion of LA to the PLL–SeNPs reduced the ζ potential and the positive charges on the NP, which could be attributed to the masking of positive charges of the PLL by the LA. The nanocomplexes all possessed low negative ζ potentials, which may be due to the nature of the pDNA conformation in the nanocomplex. It was noted that nanocomplexes <100 nm in size and with low zeta potentials (close to zero) were able to target the hepatocytes [49] successfully. These nanocomplexes did show the ability to target the HepG2 cells in vitro. The PDI provides information on the size uniformity of the NPs, with PDI values below 0.1 being an indication of a monodisperse sample population. In contrast, PDI values greater than 0.4 indicate a polydisperse sample with a higher tendency to aggregate [51]. Hence, these FSeNPs and their respective nanocomplexes were monodisperse with a low tendency to agglomerate.

Both PLL–SeNP and LA–PLL–SeNPs could efficiently bind and compact the pDNA, as evidenced by the electrophoretic mobility shift and dye displacement assays. The cationic nature of the PLL allowed for the electrostatic interaction of the anionic phosphate backbone of the pDNA with the protonated terminal lysine residues [52]. This efficiently bound and compacted the pDNA and prevented its migration through the agarose gel. As the NP concentration increased, a point of electroneutrality was reached where the negative charges of the pDNA were completely neutralized by the positive charges of the NPs. This neutral state indicated the optimum binding ratio of the pDNA to the NP. A greater amount of the LA–PLL–SeNP than PLL–SeNP was required to bind the same amount of pDNA. As mentioned above, the LA may have shielded some of the positive charges of the PLL, leading to a reduction in the binding affinity of the targeted nanocomplex for the pDNA.

The ability of NPs to compact nucleic acids is important for gene delivery applications. Ethidium bromide (EB) is a fluorescent dye that intercalates between nucleic acids’ bases. It is important to note that the dye displacement assay provides information on the amount of the NP required to compact the pDNA, whereas the electrophoretic mobility shift assay indicates the minimum amount of the FSeNP needed to bind the pDNA. Although the targeted NPs produced a higher fluorescence decay than the untargeted NPs, there was a considerable difference in the amount of NPs needed to displace the EB. The targeted NPs required a higher amount than the untargeted NPs. This trend was also observed in the electrophoretic mobility shift assay. A lower concentration of the untargeted NPs may be needed since a longer segment of PLL can progressively condense more pDNA [33]. The lower compaction of pDNA by the PLL–SeNP may be due to the conformation of the PLL encapsulating the SeNP. Since PLL may have existed primarily as β-sheets on the surface of the NP, it may have influenced the pDNA condensation as β-sheet conformations of PLL bound to NPs tend to aggregate [24,27,53].

The FSeNPs were also capable of protecting the pDNA from degradation. However, most of the pDNA remained well-bound despite the use of SDS to liberate the pDNA. Similar results were found where SDS could not fully release the DNA from the nanocomplex were reported [34,54,55]. The high compaction ability of the pDNA by the FSeNPs may have contributed to the inability of the SDS to release the pDNA from the nanocomplex fully. Hence, the pDNA remained in the wells. 

The nanocomplexes further showed low or no cytotoxicity in vitro. Se is metabolized in the liver, accounting for the HepG2 cells displaying the highest cell viability of more than 80%. Furthermore, the kidney plays a role in the metabolism and excretion of seleno-species [17,38,41,43,56,57], which could explain the high cell viability of the HEK293 cells, as well. The cytotoxic effects of SeNPs have been previously reported [22] and may be due to the concentration of sodium selenite. In the current study, a lower concentration of sodium selenite was used (0.005 M), resulting in negligible cytotoxic effects. The cytotoxicity of the SeNPs is also dependent on the concentration used [17,56,58,59]. Since low concentrations of the FSeNPs were required to bind the pDNA fully, no significant cytotoxicity was observed for the targeted and nontargeted SeNPs in all the three cell lines

The luciferase gene derived from the firefly (*Photinus pyralis*) was used to evaluate transfection activity based on the evaluation of protein produced. Luminescence produced from the protein’s (luciferase enzyme) reaction with the substrate luciferin are measured and taken as being directly proportional to the concentration of the luciferase enzyme present [57]. This is directly related to the number of cells that have been successfully transfected. The nanocomplex size can influence the cellular uptake efficiency as well as the pathway taken. Nanocomplexes that are between 120–150 nm in size are internalized via clathrin/caveolin mediated endocytosis [31,36,60]. Since PLL–SeNP was smaller than the LA–PLL–SeNP, it allowed for sufficient cellular uptake across all cell lines. However, due to the inclusion of the targeting moiety, LA-mediated cellular uptake via RME was achieved since the LA–PLL–SeNPs were recognized by the abundantly expressed ASGP-R on the HepG2 cells. The HepG2 cells are good models for the ASGP-R as they have been reported to possess over 225,000 ASGP-Rs per cell [61]. Wu and Wu [62] were the first researchers to demonstrate ASGP-R-mediated gene delivery to HepG2 cells. They used a delivery system that included asialoorosomucoid cross-linked to PLL. In this study, the LA-medixed uptake occurred through a specific interaction with the galactose moiety of LA and the ASGP-R, which has a high affinity for terminal galactose moieties and N-acetylgalactosamine [37,49]. To confirm RME, the HepG2 cells were incubated with excess LA (25× more than that which is present on LA–PLL–SeNP), which blocked the ASGP-Rs, preventing recognition of the targeted nanocomplexes and ultimately decreasing overall luciferase activity for the targeted nanocomplexes. The use of chitosan modified SeNPs has shown successful targeted delivery of mRNA to HepG2 cells using LA as a targeting ligand [28] and to KB cells using folate as the targeting ligand [63]. In addition, they have recently shown successful delivery of pDNA in vitro [64]. Overall, these PLL modified nanocomplexes were able to successfully bind, protect and deliver the pDNA in vitro, showing great promise in the use of these FSeNPs as nonviral gene delivery vehicles.

## 4. Materials and Methods

### 4.1. Materials 

Dialysis tubing (MWCO 12 and 120 kDa), poly-L-lysine (PLL) (75–150 kDa), sodium selenite, ascorbic acid, N-ethyl-N′-(3-dimethyl aminopropyl) carbodiimide (EDC), N-hydroxysuccinimide (NHS), lactobionic acid (LA), copper sulphate and bicinchoninic acid (BCA) were purchased from Sigma–Aldrich Chemical Co. (St. Louis, MO, USA). Tris (hydroxymethyl) aminomethane, sodium dihydrogen phosphate, ethylenediamine tetraacetic acid (EDTA), dimethyl sulfoxide (DMSO), 2-(4-(2-hydroxyethyl)-1-piperazinyl) ethane sulphonic acid (HEPES), 3-(4,5-dimethythiazol-2-yl)-2,5-diphenyl tetrazolium bromide (MTT), potassium chloride (KCl), PBS tablets (phosphate-buffered saline, 140 mM NaCl, 10 mM phosphate buffer) and ethidium bromide were purchased from Merck (Darmstadt, Germany). The Plasmid Factory (Bielefield, Germany) supplied the pCMV–*Luc* DNA. Ultrapure grade agarose powder was purchased from Bio-Rad Laboratories, Inc. (Richmond, VA, USA). Human embryonic kidney (HEK293), cervical cancer (HeLa) and hepatocellular carcinoma (HepG2) human cell lines were originally purchased from the ATCC (Manassas, VA, USA). Eagle’s Minimum Essential Medium (EMEM), trypsin-EDTA (trypsin (0.25% w/v), EDTA (0.1% w/v), antibiotics (penicillin (5000 units/mL)/streptomycin (5000 μg/mL) were supplied by Lonza BioWhittaker (Verviers, Belgium). Fetal bovine serum (FBS) was sourced from Gibco Invitrogen (Karlsruhe, Germany). The luciferase assay kit and 5× lysis buffer were purchaded from the Promega Corporation (Madison, WI, USA). Corning Incorporated (New York, NY, USA) provided all sterile consumable plasticware for tissue culture. Ultrapure (18 Mohm) water was used throughout the study.

### 4.2. Synthesis of Selenium Nanoparticles (SeNPs)

SeNPs were synthesized using an ascorbic acid reduction method [53]. Approximately 10 mL of a 5 mM sodium selenite (Na_2_SeO_3_) solution was added dropwise to 10 mL of ascorbic acid (20 mM) under continuous stirring for 30 min until a color change from clear to orange was noted. The newly formed SeNPs were then diluted to 25 mL and dialyzed (MWCO 12 kDa) against 18 MOhm water over 24 h at room temperature to remove any unreacted material.

### 4.3. Preparation of Poly-L-Lysine Encapsulated SeNPs (PLL–SeNP)

Briefly, 5 mL of sodium selenite (5 mM) was added to 10 mL of PLL (75–150 kDa), followed by the dropwise addition of 4 mL of ascorbic acid (20 mM) under constant stirring until a color change from clear to red was observed. The solution was left to stir overnight [65], then diluted to a final volume of 25 mL and dialyzed (MWCO 120 kDa), as in Section 4.2.

### 4.4. Preparation of Lactobionic Acid-Modified PLL–SeNPs (LA–PLL–SeNP)

Lactobionic acid (LA) was prepared as previously described [66], with modifications. Preparation involved the addition of 2.5 mL LA (0.05 M) to a 5 mL solution of 0.1 M 1-ethyl-3-(3-dimethyl aminopropyl)-carbodiimide (EDC) and 0.1 M N-hydroxysuccinimide (NHS) in a 1:1 ratio. Thereafter, 5 mL of PLL (75–1500 kDa) solution was added to the LA solution and left to stir for 6 h. Approximately 9 mL of already synthesized SeNP was added dropwise to the solution and stirred overnight. The solution was then diluted to a final volume of 20 mL and dialyzed, as in Section 4.2.

### 4.5. Characterization

#### 4.5.1. UV-Visible Spectroscopy 

UV-visible spectroscopy of the SeNPs and functionalized SeNPs (FSeNPs) was measured between 200–800 nm with 1 nm intervals on a JASCO V-730-UV-visible NIR Bio spectrophotometer (Tokyo, Japan).

#### 4.5.2. Fourier-Transform Infrared Spectroscopy (FTIR)

SeNPs and FSeNPs were freeze-dried before analysis. FTIR spectra were obtained using a Perkin Elmer Spectrum 100 FTIR spectrometer with a Universal Attenuated Total Reflectance Accessory (UATR) sampling accessory scanning from 4000–380 cm^−1^.

#### 4.5.3. Nanoparticle Tracking Analysis (NTA)

The hydrodynamic size, zeta potential and polydispersity index (PDI) of all NPs and nanocomplexes were determined using a Nanosight NS-500 (Malvern Instruments, Worcestershire, UK) at 25 °C. NPs were diluted at 1:40, while nanocomplexes were diluted at 1:100 in 18 Mohm water.

#### 4.5.4. Transmission Electron Microscopy (TEM)

Morphological characteristics of the NPs and nanocomplexes were visualized by transmission electron microscopy (TEM) (JEOL JEM-1010, Jeol, Tokyo, Japan). Micrographs were analysed and images captured using iTEM Soft Imaging Systems (SIS) Mega view III fitted with a side-mounted digital camera (3-megapixels). Carbon-coated copper grids (400-mesh, Ted Pella Inc. Redding, CA, USA) were dipped into each sample suspension and air-dried before viewing.

### 4.6. Electrophoretic Mobility Shift Assay 

The electrophoretic mobility or band shift assay [55] was used to determine the binding ratios between the FSeNPs and pDNA (0.25 μg/μL). Nanocomplexes were prepared by incubating varying amounts of the PLL–SeNPs and LA–PLL–SeNPs, with a constant concentration of pCMV–*Luc* DNA (0.25 μg/μL). Nanocomplexes were brought up to a volume of 10 μL using HBS and incubated for 1 h at room temperature [40], followed by the addition of 2 μL of gel loading buffer. A pDNA control was included to visualize the normal migration of naked pDNA. A 1% agarose gel containing ethidium bromide (EB) was used. Electrophoresis was conducted in TBE buffer for 90 min at 60 V. The gels were viewed using a Vacutec Syngene-G-box UV transilluminator imaging system, and images were captured using the GeneSnap software. The suboptimum, optimum and supraoptimum ratios obtained from this assay were used in further studies.

### 4.7. Dye Displacement Assay 

The ethidium bromide intercalation assay [55] was used to confirm the ability of FSeNPs to condense and compact the pDNA. Approximately 100 µg EB together with 100 μL HBS was added to a well in a black 96-well flat-bottom plate to establish the baseline fluorescence at 0%. After that, 1.2 µg pDNA was added, and the resulting fluorescence was measured and taken as 100%. Approximately 1 μL aliquot of the FSeNPs was then added to the wells and mixed, and the fluorescence was measured until a plateau was reached. A GloMax^®^-Multi Detection System (Promega BioSystems, Sunnyvale, CA, USA) was used to determine the fluorescence at 520 nm (excitation wavelength) and 600 nm (emission wavelength). The relative fluorescence was plotted against the amount of the respective functionalized SeNP used using the following equation.

F*_r_* (%) = (F_i_ − F_0_)/(F_max_ − F_0_) × 100

where F_r_ is the relative fluorescence (%), F*_i_* is the absorbance of FSeNP at a given concentration, F_0_ is the baseline fluorescence and Fmax is the fluorescence at 100%.

### 4.8. Enzyme Protection Assay 

The nuclease protection assay was conducted to assess the ability of the FSeNPs to protect the pDNA from enzymatic digestion, as previously described [55]. Nanocomplexes were prepared as described in Section 4.6. Following the incubation period, 1 μL of foetal bovine serum (FBS) was added to each sample and incubated at 37 °C for 4 h. Two controls were used, a positive control that contained pDNA in the absence of FBS and a negative control that contained pDNA treated with 10% FBS. Thereafter, 1.1 μL EDTA (10 mM) was added to each sample to inhibit the action of the FBS, followed by the addition of SDS to a final concentration of 5% to release the pDNA from the nanocomplexes. The samples were then incubated at 55 °C for 20 min and then subjected to agarose gel electrophoresis and visualized, as described in Section 4.6. 

### 4.9. MTT Cell Viability Assay 

The MTT assay was used to assess the viability of the HEK293, HeLa and HepG2 cells in the presence of the FSeNP nanocomplexes at a suboptimum, optimum and supraoptimum ratio as previously described [56]. Cells at a density of 3.5 × 10^6^ cells/well in 48-well plates were incubated overnight at 37 °C to allow the cells to attach. Thereafter, the medium was replaced, cells treated with 10 μL of the respective nanocomplex and then incubated for 48 h at 37 °C. A control of untreated cells was included, indicating 100% cell viability. All studies were conducted in triplicate. Thereafter, the medium was replaced with 200 μL of fresh medium containing 10 μL of MTT reagent (5 mg/mL in PBS) and incubated for a further 4 h at 37 °C. Medium containing MTT was then removed, and 200 μL of DMSO was added to the wells. The sample absorbance was measured using a Mindray MR-96A microplate reader (Vacutec, Hamburg, Germany) at 570 nm.

### 4.10. Luciferase Expression Assay

The transfection efficiency of each nanocomplex was assessed quantitively using the luciferase reporter gene assay, as previously described [41]. All assays were conducted in triplicate and included two controls: untreated cells and cells treated with naked pDNA. Cells were seeded, and nanocomplexes were added to the cells as described in Section 4.9. The medium was removed after the 48 h incubation, and cells were washed twice with PBS. Thereafter, 80 μL of a 1× cell lysis buffer was added to each well, and the plate was rocked on a Scientific STR 6 platform shaker (Stuart Scientific, Staffordshire, UK) for 15 min at 30 rev/min. The lysed cells were removed from the wells, transferred to microcentrifuge tubes and centrifuged at 12,000× *g* for 5 s. Approximately 20 μL of the cell-free supernatants were transferred to a white 96-well plate, into which 100 μL of the luciferase assay reagent was injected into each well. A GloMax^®^-Multi Detection System (Promega Biosystems, Sunnyvale, CA, USA) was used to measure the luminescence. Protein content was determined using a standard bicinchoninic acid (BCA) assay, and the luciferase activity was expressed as relative light units (RLU)/mg protein. 

### 4.11. Competition Binding Assay 

The competition binding assay was conducted as described [17], with modifications. It was used to confirm the uptake of the LA–PLL–SeNP by the asialoglycoprotein receptor (ASGP-R), present on the HepG2 cells via RME. HepG2 cells were seeded and incubated as in Section 4.9. Following incubation, the medium was removed and replaced with fresh medium, together with free LA (55 mg/mL per well), which was 25× the amount of the LA coating on the LA–PLL–SeNPs. After 30 min at 37 °C, the nanocomplexes were added as in Section 4.9, and cells were incubated for 48 h, followed by the luciferase reporter gene assay as described in Section 4.10.

### 4.12. Apoptosis 

Any apoptosis induced due to the nanocomplexes at the optimal binding ratio was determined using the dual acridine orange (100 μg/mL)/ethidium bromide (100 μg/mL) (AO/EB) staining, as previously described [40]. Cells were prepared as in Section 4.9. After 24 h incubation, the cells were treated with the respective nanocomplexes. An untreated positive cell control was included. Cells were then incubated for 48 h at 37 °C, followed by the removal of media and washing of the cells with PBS. Thereafter, 10 μL of the AO/EB solution was added to the cells, and the plate was rocked on a Scientific STR 6 platform shaker (Stuart Scientific, Staffordshire, UK) for 5 min at 30 rev/min. Cells were washed with PBS and viewed under an Olympus fluorescence microscope fitted with a CC12 fluorescence camera (Olympus Co., Tokyo, Japan). The apoptotic index for each cell line was calculated using the following equation:

Apoptotic index = (number of cells)/(total number of cells counted)

### 4.13. Statistical Calculations

All assays were conducted in triplicate. Data are presented as mean ± standard deviation (*n* = 3). Statistical analysis between means was conducted using multiple comparisons grouped two-way analyses of variants (ANOVA) using the statistical software programme GraphPad Prism version 6.01 (GraphPad Software, La Jolla, CA, USA). * *p*-value < 0.05, ** *p*-value < 0.01 and **** *p*-value < 0.0001 were considered significant. 

## 5. Conclusions

This study has revealed that the synthesized FSeNP formulations were stable, biocompatible and capable of efficient encapsulation and safe intracellular delivery of pDNA. The FSeNP nanocomplexes displayed nanoscale sizes, afforded protection to the pDNA cargo, exhibited negligible cytotoxicity with low apoptotic indices and enhanced transfection efficiencies. The inclusion of LA into the SeNP formulation allowed for cell specificity through active targeting with enhanced transfection observed in the ASGP-R rich HepG2 cells treated with the LA–PLL–Se nanocomplexes. Overall, these FSeNP nanocomplexes show great promise for their use as gene delivery vehicles and warrant in vivo studies to ascertain their ultimate potential as a treatment strategy for liver cancer. 

## Figures and Tables

**Table 1:** Nanoparticle and nanocomplexe sizes, zeta potential and polydispersity index (PDI) from NTA. Data are presented as means ± SD (*n* = 10).

Nanoparticles				Nanocomplexes			
	Size (nm)	ζ Potential (mV)	PDI	pDNA:NP Ratio (w/w)	Size (nm)	ζ Potential (mV)	PDI
SeNP	75.7 ± 0.8	−12.1 ± 0.2	0.00011	-	-	-	-
PLL–SeNP	84.7 ± 10	28.6 ± 10	0.014	1:2.7	118.7 ± 16.3	−26.9 ± 0.6	0.0189
LA–PLL–SeNP	124.3 ± 3.2	25.0 ± 6.3	0.00066	1:14	164.5 ± 77	−21.1 ± 0.3	0.0219

**Table 2:** Suboptimal, optimal and supraoptimal binding ratios of the FSeNP:pDNA.

Nanocomplex	Suboptimal Ratio (w/w)	Optimal Ratio (w/w)	Supraoptimal Ratio (w/w)
PLL–SeNP:pDNA	1:2.2	1:2.7	1:3.2
LA–PLL–SeNP:pDNA	1:10.9	1:14	1:16

**Table 3:** Apoptotic indices of FSeNP nanocomplexes at their optimum binding ratios.

Cell Line	Apoptotic Index	
	PLL–SeNP	LA–PLL–SeNP
HEK293	0.04	0.09
HeLa	0.08	0.07
HepG2	0.13	0.12

## Data Availability

All data and contributions presented in the study are included in the article. Further information can be obtained from the corresponding author.

## Abbreviations

AO	Acridine orange
ASGP-R	Asialoorosomucoid receptor
BCA	Bicinchoninic acid
DMSO	Dimethyl sulfoxide
EB	Ethidium bromide
EDC	N-ethyl-N′-(3-dimethyl aminopropyl) carbodiimide
EDTA	Ethylenediamine tetraacetic acid
EMEM	Eagle’s Minimum Essential Medium
FBS	Fetal bovine serum
FLC	Fibrolamellar HCC
FSeNPs	Functionalized SeNPs
FTIR	Fourier-transform infrared spectroscopy
HBS	HEPES buffered saline
HCC	Hepatocellular carcinoma
HCC-CCA	Mixed hepatocellular cholangiocarcinoma
HEPES	2-(4-(2-hydroxyethyl)-1-piperazinyl) ethane sulphonic acid
iCCA	Intrahepatic cholangiocarcinoma
KCl	Potassium chloride
LA	Lactobionic acid
LA–PLL–SeNP	Lactobionic acid-modified Poly-L-lysine encapsulated SeNPs
mRNA	Messenger RNA
MTT	(3-(4,5dimethylthiazol-2-yl)-2,5-diphenyltetrazolium bromide
NHS	N-hydroxysuccinimide
NPs	Nanoparticles
NTA	Nanoparticle tracking analysis
PBS	Phosphate-buffered saline
PDI	Polydispersity index
pDNA	Plasmid DNA (Specifically referring to pCMV-*Luc* DNA in this study).
PLL	Poly-L-lysine
PLL–SeNP	Poly-L-lysine encapsulated SeNPs
RLU	Relative light units
RME	Receptor-mediated endocytosis
SDS	Sodium dodecyl sulphate
Se	Selenium
SeNPs	Selenium nanoparticles
SPR	Surface plasmon resonance
TBE	Tris-Borate-EDTA
TEM	Transmission electron microscopy
UV-vis	UV-visible spectroscopy
